# The American Ophthalmological Society

**Published:** 1866-08

**Authors:** 


### BOSTON CORRESPONDENCE.

### THE AMERICAN OPHTHALMOLOGICAL SOCIETY.

Boston, June 19, 1866.

The habits of life, formed during a vacation spent in visiting the friends and scenes of one's early days, are not conducive to mental effort, even to the extent of writing a letter.

My interest, however, in the meetings of the American Ophthalmological Society, which have just been held in this city, and the thought that a short account of them might be acceptable to the readers of the Journal, have induced me to take the pen for the moment.

The opening meeting of the society was held at the Massachusetts Charitable Eye and Ear Infirmary, Tuesday, June 12th. Twenty-two members were present, all specially interested in ophthalmology, from Boston, New York, Philadelphia, Baltimore, Albany, Cincinnati and Chicago.

The Chairman of the society was the venerable Edward Delafield, M. D., who, fifty years ago, commenced with great enthusiasm the study of ophthalmology, and at that early period devoted much of his energies to the establishment of the Charitable Eye Infirmary in New York, now one of the most important charities of the city.

Undoubtedly, the presence of such a chairman enhanced the dignity with which the discussions were conducted. The society has been most fortunate in securing a president so full of wisdom, so aged, and yet so full of vigor and interest for the welfare of the society and the cultivation of ophthalmology in America.

The proceedings will soon be published, when you can select for your readers such articles as may seem of particular interest.

I am confident the annual meetings of this association will directly and indirectly accomplish much good. There will be no small advantage and pleasure enjoyed by each member in becoming personally acquainted with educated men from different cities of the Union, devoted to the study of ophthalmic medicine and surgery.

The interchange of views, and the history of different personal experiences, cannot be without profit. The association must inevitably exert also a good influence upon the medical profession at large — must tend to increase interest in the culture of ophthalmology, and remove the prejudice against professed oculists, even now existing to a certain extent in the profession.

Judging from what I heard at the sessions, at the social gatherings, and in private conversation with individuals, I believe the members, perhaps without exception, are determined by their devotion to the study of their specialty, by scrupulously avoiding even the appearance of charlatanry, to command the best respect of the whole profession of medicine. I think advertisements in medical journals will be wholly discontinued by all members of the society. There is, in fact, almost an unanimous disposition to frown upon the slightest infringement on the spirit as well as the letter of the code of ethics.

It is certainly very unjust for the profession to hold specialists responsible for the acts of men who practice them unworthily—as unjust as it would be for the public to hold the medical profession responsible for every kind of quackery.

Oculists are undoubtedly inclined to be enthusiastic—possibly sometimes too enthusiastic in their estimation of the relative importance of their special department. But when we consider the incalculable value of the organ they endeavor to preserve, and look upon the splendid discoveries of the ophthalmoscope and of the operation of iridectomy; when we study the truths but recently established, respecting the function of accommodation of vision at different distances and its anomalies—the physiology of the oblique muscles, and the modifications in several important operations—we can overlook this weakness, if weakness it is.

And the oculist may be excused for pointing with pride to the many names which have honored his specialty, to the character of the ophthalmological societies in other countries, and of the papers which their members have produced, to the standard works upon diseases of the eye, and to the great merit of the periodicals devoted to the interests of ophthalmic science.

The social features of the convention should not be forgotten.

Dr. J. H. Dix, one of the oldest oculists in the city of Boston, gave an evening entertainment at his residence, not only to the members of the society, but also to many distinguished physicians of Boston and its vicinity.

Everything that could please, was afforded the guests of the evening—most delightful music, exquisite flowers in profusion, a table richly loaded with all that taste could suggest, and an opportunity for familiar conversation with many of the best minds in the profession.

On the following evening, the members of the society residing in Boston, gave a splendid banquet at the Parker House, at which only members of the society were present.

Beautiful flowers, elegant dishes of every kind, with delicate fruits in superabundance, and arranged in perfect taste, left nothing for the eye or appetite to desire. After all material wants, stimulated by the tempting delicacies of the table, had been more than satisfied, a couple of hours were passed in pleasant exchange of sentiment and good wishes.

You can be assured we all left Boston pleased with the success of our meeting, and grateful to our friends for their most successful efforts to entertain us.

The next meeting of the society will be held at Niagara, in June of 1867. E. L. H.

